# Arginine ingestion inhibits phagocyte invasion in eccentrically contracted rat fast-twitch muscle

**DOI:** 10.1007/s10974-024-09672-w

**Published:** 2024-04-18

**Authors:** Keita Kanzaki, Masanobu Wada

**Affiliations:** 1 Department of Clinical Nutrition, Faculty of Health Science and Technology, Kawasaki University of Medical Welfare, 288 Matsushima, Kurashiki, Okayama 701-0193 Japan; 2 Graduate School of Humanities and Social Sciences, Hiroshima University, 1-7-1 Kagamiyama, Higashihiroshima, Hiroshima 739-8521 Japan

**Keywords:** Nitric oxide, Nitric oxide synthase, Inflammation, Muscle damage

## Abstract

Eccentric contraction (ECC) has been shown to induce leukocyte invasion into skeletal muscle, resulting in muscle inflammation. This study aimed to investigate whether prior ingestion of L-arginine (ARG), a nitric oxide precursor, inhibits ECC-induced macrophage invasion. Male Wistar rats received ARG in water for 7 days, beginning 3 days prior to ECC. ECCs were induced in the anterior crural muscles for 200 cycles. Three days later, the tibialis anterior and extensor digitorum longus muscles were excised for biochemical analysis and force measurement, respectively. ARG ingestion increased nitrite and nitrate levels in plasma and muscle, inhibiting force depression and reducing CD68 content in muscles subjected to ECC. ARG ingestion also ameliorated an ECC-induced increase in protein nitration, although neither ARG ingestion nor ECC induction affected protein carbonyl levels. The present results suggest that ingestion of ARG or ARG-rich foods may alleviate inflammation by attenuating phagocyte invasion in eccentrically contracted skeletal muscles.

## Introduction

Combined isometric, concentric, and eccentric muscle contractions are integral to daily activities. Repeated contractions result in muscle fatigue, defined as a gradual decrease in force (Allen et al. 2008). Although force typically recovers rapidly during the recovery period, contractions involving a substantial eccentric component often lead to prolonged force depression, which requires several days of recovery. This is primarily attributed to muscle damage characterized by myofibrillar integrity loss, excitation–contraction coupling failure, increased sarcolemmal permeability (membrane damage), muscle swelling, and delayed-onset muscular soreness (Hody et al. 2019; Kanzaki et al. 2022).

Nitric oxide (NO) is produced by NO synthase (NOS) from L-arginine (ARG), NADPH, and oxygen (Ghimire et al. 2017). Three mammalian NOS isoforms exist: neural NOS (nNOS), endothelial NOS (eNOS), and inducible NOS (iNOS). Skeletal muscle expresses all three isoforms, of which nNOS and eNOS are constitutive isoforms. At rest, NO is produced in muscles primarily by nNOS. Modest NO increases play a critical role in cellular function. In skeletal muscle, NO modulates ryanodine receptor, a sarcoplasmic reticulum Ca^2+^ release channel, and glucose uptake, as well as maintaining mitochondrial function (Allen et al. 2016). However, excessive NO production leads to *S*-nitrosylation through its reaction with cysteine thiol to form *S*-nitrosothiol. Additionally, the interaction between NO and superoxide forms peroxynitrite, a potent oxidant that can modify lipids and proteins.

Neutrophils and macrophages, inflammatory cells that release superoxide and NO, respectively, invade the intracellular space of muscles subjected to eccentric contraction (ECC; hereafter, referred to as “ECC muscles”). The invasion causes inflammation, potentially contributing to ECC-induced force depression (Lapointe et al. 2002). Thus, blocking inflammatory cell invasion would be expected to suppress muscle damage and subsequent force depression in ECC muscles. Indeed, a previous study has shown that ECC-induced force depression is attenuated in β2 integrin CD18 knockout mice (neutrophil adhesion to vascular endothelium, a prerequisite for invasion, is mediated by CD18) (Pizza et al. 2005).

Despite extensive research, the mechanisms underlying inflammatory cell infiltration in ECC muscles remain incompletely understood. Although ECC increases membrane damage, evidenced by increased serum creatine kinase activity, membrane damage is unlikely to cause inflammatory cell invasion, as invasion precedes changes in serum creatine kinase activity (Peake et al. 2017). Studies on *mdx* mice, a model of Duchenne muscular dystrophy exhibiting chronic muscle inflammation, have demonstrated that intraperitoneal injection of L-arginine (ARG), the precursor of NO, decreases macrophage-secreted proinflammatory cytokine level through a NOS-mediated process (Voisin et al. 2005; Hnia et al. 2008). These findings highlight the potential for ARG treatment to ameliorate inflammatory cell invasion into ECC muscles; however, this possibility has not been investigated.

This study aims to elucidate the effects of ARG ingestion on the invasion of phagocytes (i.e. macrophages and neutrophils) into ECC muscles. We hypothesize that ARG ingestion prior to ECC alleviates phagocyte invasion and protein nitration. To test this hypothesis, rats were administrated ARG for 7 days, starting 3 days before the ECC protocol. Experiment results from fast-twitch muscles supported our hypothesis.

## Methods

### Ethical approval and animal care

All procedures used in this study received approval from the Institutional Animal Care and Use Committee of Kawasaki University of Medical Welfare (No. 17-012). Eighteen male Wistar rats (6 weeks old) were purchased from Charles River Laboratory (Yokohama, Japan). They were given water and standard chow (MF; Oriental Yeast Corporation, Tokyo, Japan) containing 1.53% (mass/mass) ARG ad libitum and housed in a thermally controlled room at 22–26 °C with a 12/12 h light/dark cycle for at least 1 week before the experiment began. At the end of the experiments, the rats were euthanized with an overdose of isoflurane, followed by cervical dislocation.

### ARG ingestion and ECC protocol

Rats aged 7–8 weeks old (~ 290 g) were randomly assigned to a control (CON) or ARG group (n = 9 in each group). ARG rats were received water containing 0.4% (mass/vol.) ARG (FUJIFILM Wako Pure Chemical Corporation, Osaka, Japan) ad libitum for 7 days, starting 3 days before and continuing for 3 days after the ECC protocol. The mean ARG intake in this study (584 mg/kg body wt/day) aligned with previous studies using similar protocols (Lomonosova et al. 2014; Kanzaki et al. 2018). CON rats received water without ARG.

Under anesthesia, achieved with an intraperitoneal injection of medetomidine (0.4 mg/kg body wt), midazolam (2.0 mg/kg body wt), and butorphanol (2.5 mg/kg body wt), rats were placed in a supine position, and the left foot was secured in a homemade foot holder attached to the rim of a servomotor. ECC was induced using an electrical stimulator (SEN-3401; Nihon Kohden, Tokyo, Japan) as previously described (Kanzaki et al. 2017, 2018). Briefly, ECCs were elicited in the anterior crural muscles by stimulating the peroneal nerve with a 1/s train of 1/ms pulses at 50 Hz and supramaximal voltage during forced plantar flexion (150° angular movement at 150°/s from 30° ankle flexion). ECC was repeated every 4 s for 200 cycles. Anesthetized rats were recovered using an intraperitoneal injection of the medetomidine antagonist atipamezole (0.8 mg/kg body wt).

Three days after ECC induction, the extensor digitorum longus (EDL) and tibialis anterior (TA) muscles were excised from both legs under anesthesia. Muscles from the contralateral limb served as resting controls. Tail vein blood was collected, drawn into a tube containing EDTA, and centrifuged at 1000 g and 4 °C for 10 min. Both the obtained plasma and minced TA muscles were stored at −80 °C until analyses.

### Isometric force measurement

To eliminate potential influences of acute muscle contractions on subsequent analyses, EDL and TA muscles were used for force measurements and biochemical analyses, respectively. Isolated EDL muscles were mounted between two platinum plate stimulation electrodes (Iwashiya Kishimoto Medical Instruments, Kyoto, Japan) and connected to an isometric force transducer (TB-611 T; Nihon Kohden). The muscles were placed in Krebs–Ringer solution [in mM: 115 NaCl, 20 NaHCO_3_, 11 glucose, 5 KHCO_3_, 5 *N*,*N*-bis(2-hydroxyethyl)-2-aminoethanesulfonic acid, 2 CaCl_2_, 1 MgCl_2_, 0.4 glutamine, and 0.3 glutamic acid] with continuous bubbling of 95% O_2_-5% CO_2_, maintaining an extramuscular pH of 7.4. Isometric contractions were elicited via direct stimulation at 10 and 80 Hz with supramaximal voltage, 1/ms pulses, and 1/s trains. Force output was recorded on a personal computer and analyzed using LabChart version 8 (ADInstruments, CO, USA). All measurements were performed in a temperature-controlled room at 25 °C. Absolute force was normalized to cross-sectional area, calculated as wet muscle weight divided by the product of muscle length and density (1.06 mg/mm^3^).

### Nitrite/nitrate concentration measurement

Nitrite and nitrate (NOx) concentrations were determined following established protocols (Kanzaki et al. 2018, 2019). Frozen TA muscles (~ 150 mg) were pulverized under liquid nitrogen, vortexed with 4 vol. (vol./mass) of ice-cold phosphate-buffered saline (pH 7.4) containing 10 mM *N*-ethylmaleimide and 2.5 mM EDTA, and centrifuged at 10,000 g and 4 °C for 5 min. The resultant supernatant or thawed plasma was mixed with Griess reagent, and absorbance at 540 nm was measured after a 30-min incubation at 37 °C.

### Immunoblot analysis

TA muscles (~ 150 mg) were homogenized in 9 vol. (vol./mass) of an ice-cold homogenization buffer [100 mM KCl, 20 mM Tris/HCl (pH 7.4), 5 mM EDTA, 5 mM *N*-ethylmaleimide, 0.05% (vol./vol.) Triton X-100, and protease inhibitor cocktail (25,955-11; Nacalai Tesque, Kyoto, Japan)]. In preliminary experiments, we noted that the eNOS monomer was identified solely in the supernatant, not in the whole muscle homogenate. Therefore, one third of the homogenate was centrifuged at 10,000 g and 4 °C for 15 min, and the resulting supernatant was collected. Protein content of the supernatant and homogenate was determined using the Bradford assay, with bovine serum albumin serving as a standard (Bradford 1976).

Samples were diluted with a sample buffer [62.5 mM Tris/HCl (pH 6.8), 2% (mass/vol.) SDS, 10% (vol./vol.) glycerol, 10% (vol./vol.) 2-mercaptoethanol, and 0.02% (mass/vol.) bromophenol blue] and heated for 5 min at 95 °C. Subsequently, they were subjected to either 6% (for nNOS and eNOS monomer) or 10% (for CD68, NADPH oxidase 2, and protein carbonyl) SDS-PAGE. For analyses of nNOS dimer, eNOS dimer, and 3-nitrotyrosine, samples were diluted in the sample buffer without 2-mercaptoethanol but not heated before undergoing 6% low-temperature SDS-PAGE (for nNOS and eNOS dimer) (Klatt et al. 1995) or 10% SDS-PAGE (for 3-nitrotyrosine). Proteins were transferred to polyvinylidene difluoride membranes, which were blocked with Tris-buffered saline (pH 7.4) containing 5% (mass/vol.) skim-milk and 0.1% (vol./vol.) Tween-20 for 60 min at room temperature. Subsequently, the membranes were incubated overnight at 4 °C with the following primary antibodies: anti-CD68 (1:1,000 dilution; ab31630, Abcam, Cambridge, UK), anti-eNOS (1:150 dilution; SC-376751, Santa Cruz Biotechnology, TX, USA), anti-3-nitrotyrosine (1:500 dilution; ab52309, Abcam), anti-nNOS (1:500 dilution; sc-5302, Santa Cruz Biotechnology), and anti-NADPH oxidase 2 (NOX2) catalytic subunit gp91^phox^ (1:500 dilution; sc-130543, Santa Cruz Biotechnology). For protein carbonyl analysis, the membranes were reacted with dinitrophenylhydrazine before the blocking step, followed by a 60 min incubation at room temperature with an antidinitrophenyl antibody (1:2000 dilution; #ROIK03, Shima Laboratories, Tokyo, Japan). The membranes were then incubated for 60 min at room temperature with anti-mouse (1:7500 dilution; P0260, Dako, Glostrup, Denmark) or anti-rabbit (1:7500 dilution; sc-2357, Santa Cruz Biotechnology) secondary antibodies.

Finally, target proteins were visualized using ImmunoStar Zeta (FUJIFILM Wako Pure Chemical Corporation), and images were captured using LumiCube Plus (Liponics, Tokyo, Japan). To quantify total proteins on membranes, they were stained with Coomassie Blue R or Ponceau S. Densitometric analysis of immunoreactive and total proteins was performed using Image J (National Institute of Health, MD, USA), and the target protein amount was normalized to total proteins.

### Statistical analysis

Values were represented as means ± SD. Student’s *t*-tests were used to compare changes in plasma NOx content. Effects of ARG ingestion (CON vs. ARG rats) and ECC (rested vs. ECC muscles) were assessed using two-way repeated-measures ANOVA. Regarding CD68, NOX2, and 3-nitrotyrosine, rank transformation was applied before ANOVA owing to nonnormality and heteroscedasticity in the data. When significant differences were found, Holm–Sidak post hoc tests were performed. The significance level was set at *P* < 0.05. Statistical analyses were performed using SigmaPlot version 14.5 (Systat software, CA, USA).

## Results

### Force output

**Fig. 1:**
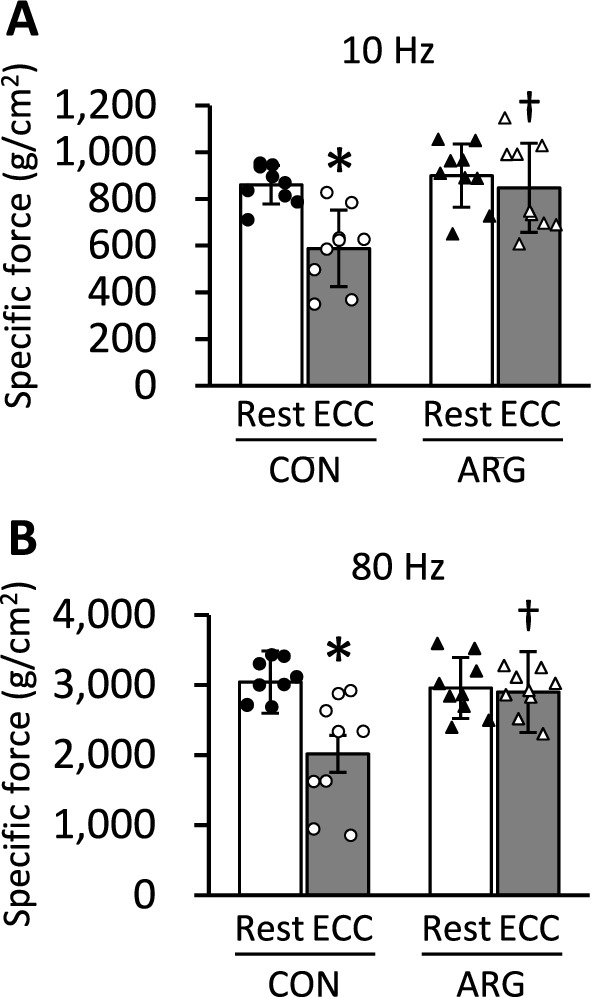
Effects of ARG ingestion and ECC induction on force output at 10 Hz (**A**) and 80 Hz (**B**) in extensor digitorum longus muscle. ARG rats were provided water containing 0.4% ARG ad libitum for 7 days starting 3 days before ECC. ECCs were elicited in the anterior crural muscles for 200 cycles. The resting muscles of the contralateral legs were used as controls. Three days after ECC, the extensor digitorum longus and tibialis anterior muscles were excised and used for force measurement and biochemical analysis, respectively. Values are means ± SD (n = 9 for each group). *P < 0.05, vs. resting muscles within rats (two-way repeated-measures ANOVA, followed by Holm–Sidak post hoc tests); ^♰^*P* < 0.05, vs. ECC muscles from CON rats (two-way repeated-measures ANOVA, followed by Holm–Sidak post hoc tests). ARG, L-arginine; CON, control; ECC, eccentric contraction

A significant interaction between ARG ingestion and ECC induction was observed for force output at both 10 Hz (*P* = 0.001) and 80 Hz (*P* = 0.006; Fig. 1). In CON rats, forces at 10 and 80 Hz in ECC muscles were 68% (*P* < 0.001) and 66% (*P* < 0.001) of those in resting muscles, respectively. Conversely, such differences between resting and ECC muscles were not observed in ARG rats. Forces in ECC muscles were significantly higher in ARG rats than in CON rats (*P* < 0.05).

### NOx concentration

**Fig. 2:**
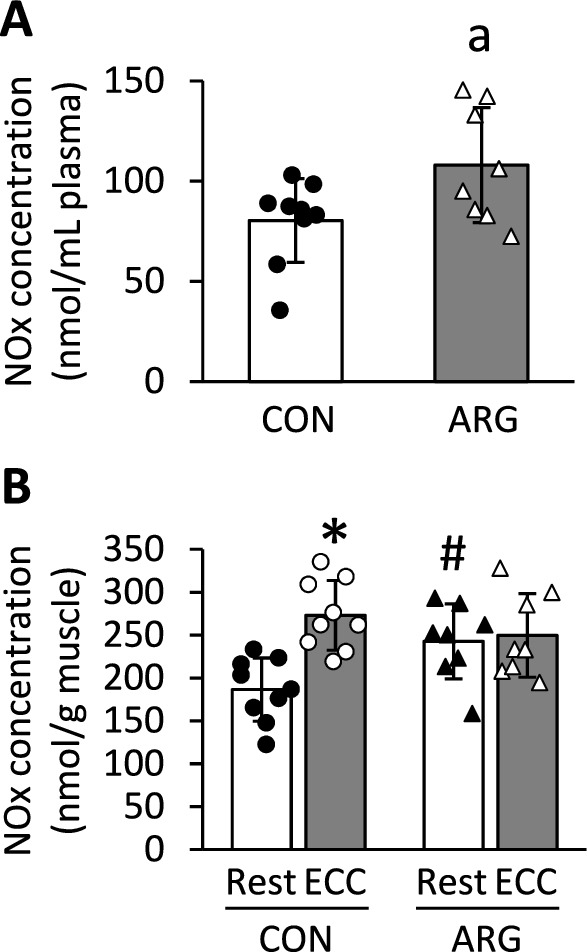
Effects of ARG ingestion and ECC induction on NOx concentration in plasma (**A**) and tibialis anterior muscle (**B**). For the experimental protocols, see the legend of Fig. 1. Values are means ± SD (n = 8–9 for each group). ^a^*P* < 0.05, vs. CON rats (Student’s *t*-tests). *P < 0.05, vs. resting muscles within rats (two-way repeated-measures ANOVA, followed by Holm–Sidak post hoc tests); ^#^*P* < 0.05, vs. resting muscles from CON rats (two-way repeated-measures ANOVA, followed by Holm–Sidak post hoc tests). ARG, L-arginine; CON, control; ECC, eccentric contraction; NOx, nitrite and nitrate

Plasma NOx concentration was significantly higher in ARG rats than in CON rats (*P* = 0.037; Fig. 2A). Regarding muscle NOx, a significant interaction was found between ARG ingestion and ECC induction (*P* = 0.010; Fig. 2B). In CON rats, NOx concentration in ECC muscles increased to 137% of that in resting muscles (*P* < 0.001), whereas such differences were not observed in ARG rats. NOx concentration in the resting muscles of ARG rats amounted to 124% of that in the resting muscles of CON rats (*P* = 0.011).

### CD68 and NOX 2 content

**Fig. 3:**
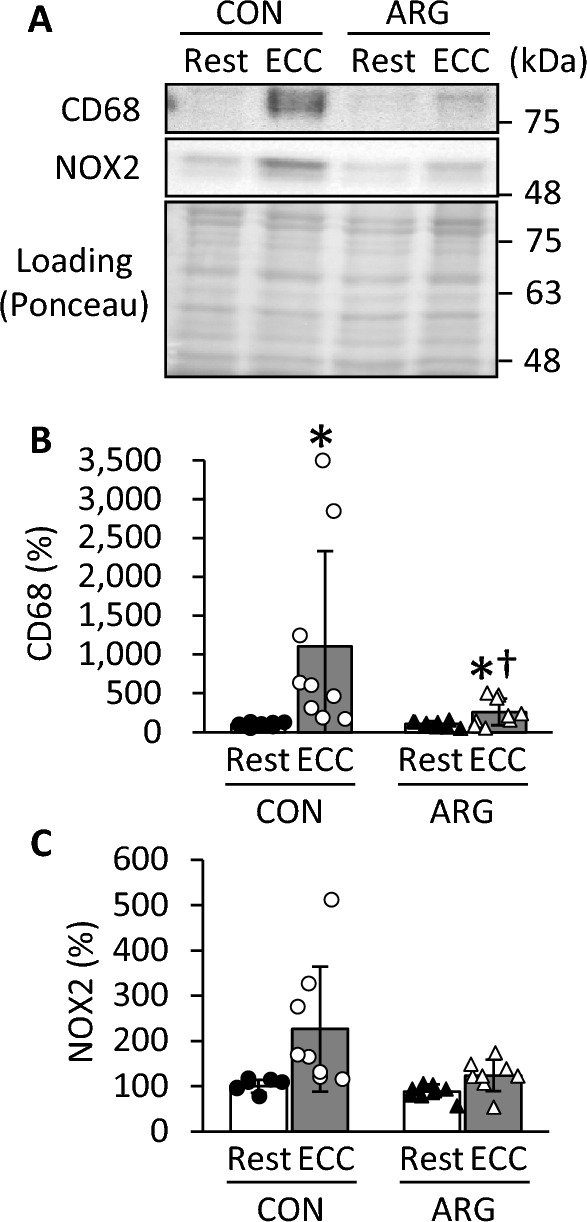
Effects of ARG ingestion and ECC induction on inflammatory macrophage (CD68) and NADPH oxidase 2 (NOX2) contents in tibialis anterior muscle. For the experimental protocols, see the legend of Fig. 1. **A** representative immunoblots of CD68 and NOX2. Apparent molecular masses are indicated on the right side. For total protein quantification, the membrane was stained with Ponceau S. **B** and **C** contents of CD68 and NOX2, respectively. Values are means ± SD (n = 8–9 for each group). The contents are normalized to total proteins. The results are expressed as percentages of the values in resting muscles from CON rats. For NOX2, the main effects were that the content was higher in ECC than in resting muscles (*P* < 0.001) and was lower in ARG than in CON rats (*P* = 0.035; rank transformation, followed by two-way repeated-measures ANOVA). *P < 0.05, vs. resting muscles within rats (rank transformation, followed by two-way repeated-measures ANOVA and Holm–Sidak post hoc tests); ^♰^*P* < 0.05, vs. ECC muscles from CON rats (rank transformation, followed by two-way repeated-measures ANOVA and Holm–Sidak post hoc tests). ARG, L-arginine; CON, control; ECC, eccentric contraction

Although CD68 is present in pro-inflammatory macrophages (M1), anti-inflammatory macrophages (M2), and neutrophils (Damoiseaux et al. 1994; Amanzada et al. 2013; Sloboda et al. 2018), only a small amount of M2 macrophages is present in the muscle cell in the early stages of inflammation (Peake et al. 2017; Sloboda et al. 2018). Therefore, CD68 contents can be used as an approximation of M1 and neutrophil levels. A significant interaction between ARG ingestion and ECC induction was observed in CD68 content (*P* = 0.042; Fig. 3A, B). In CON rats, CD68 content in ECC muscles increased to ∼11-fold of that observed in resting muscles (*P* < 0.001). In ARG rats, CD68 content in ECC muscles increased to ∼2.5-fold of that in resting muscles (*P* = 0.004) and was significantly lower than that in the ECC muscles of CON rats (*P* = 0.011). Regarding NOX2 content, no significant interaction was observed between ARG ingestion and ECC induction (Fig. 3C). The main effects included higher NOX2 content in ECC muscles than in resting muscles (*P* < 0.001) and lower NOX2 content in ARG rats than in CON rats (*P* = 0.035).

### nNOS and eNOS content

**Fig. 4:**
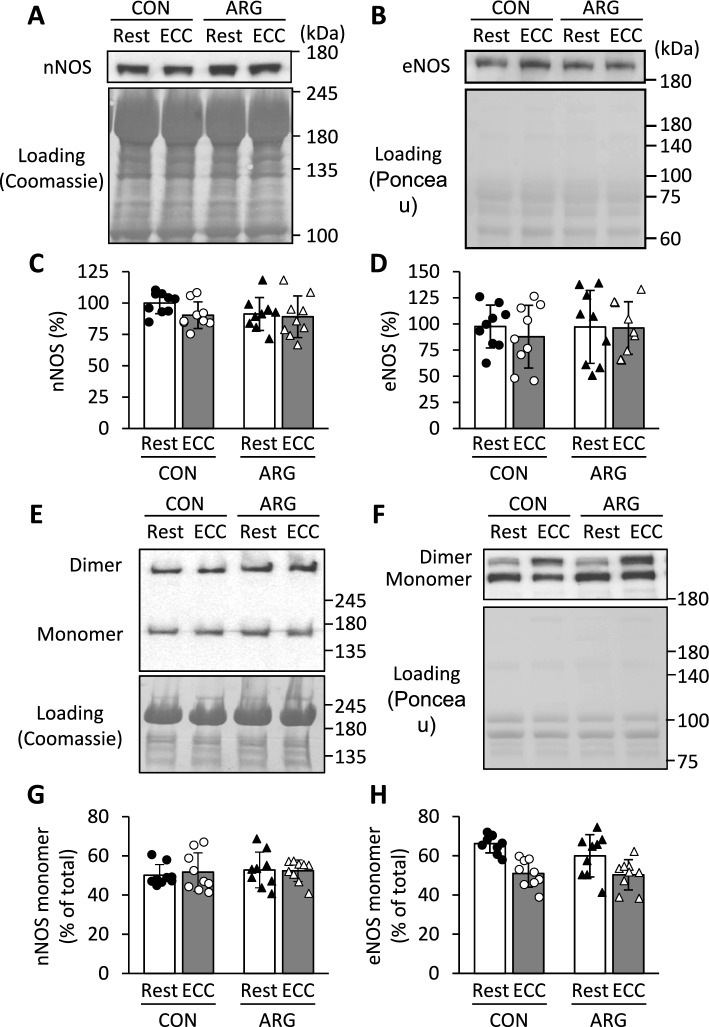
Effects of ARG ingestion and ECC induction on nNOS and eNOS contents in tibialis anterior muscle. For the experimental protocols, see the legend of Fig. 1. **A** and **B** representative immunoblots of nNOS and eNOS under normal denaturing conditions, respectively. Apparent molecular masses are indicated on the right side. **C** and **D** contents of nNOS and eNOS, respectively. The contents are normalized to total proteins. The results are expressed as percentages of the values in resting muscles from CON rats. **E** and **F** representative immunoblots of nNOS and eNOS using low-temperature SDS-PAGE, respectively. **G** and **H** proportion of monomeric nNOS and eNOS, respectively. The proportion is expressed as a percentage of the total content (monomer + dimer). For total protein quantification, the membrane was stained with Coomassie blue R or Ponceau S. For eNOS monomer, the main effects of ECC induction were observed (Rest > ECC; two-way repeated-measures ANOVA). Values are means ± SD (n = 9 for each group). ARG, L-arginine; CON, control; ECC, eccentric contraction; eNOS, endothelial nitric oxide synthase; nNOS, neuronal nitric oxide synthase

Monomers and dimers of nNOS and eNOS exist, with monomers unable to produce NO (Andrew and Mayer 1999). Neither ARG ingestion nor ECC induction affected the total content of both isoforms (Fig. 4A–D) or the proportion of monomers to total nNOS (Fig. 4E, G). Conversely, the main effect was a lower proportion of eNOS monomers in ECC muscles than in resting muscles (Fig. 4F, H), suggesting that NO synthesis via this isoform may be promoted in ECC muscles. iNOS was not detected through immunoblotting (results not shown).

### Protein carbonyl and 3-nitrotyrosine content

**Fig. 5:**
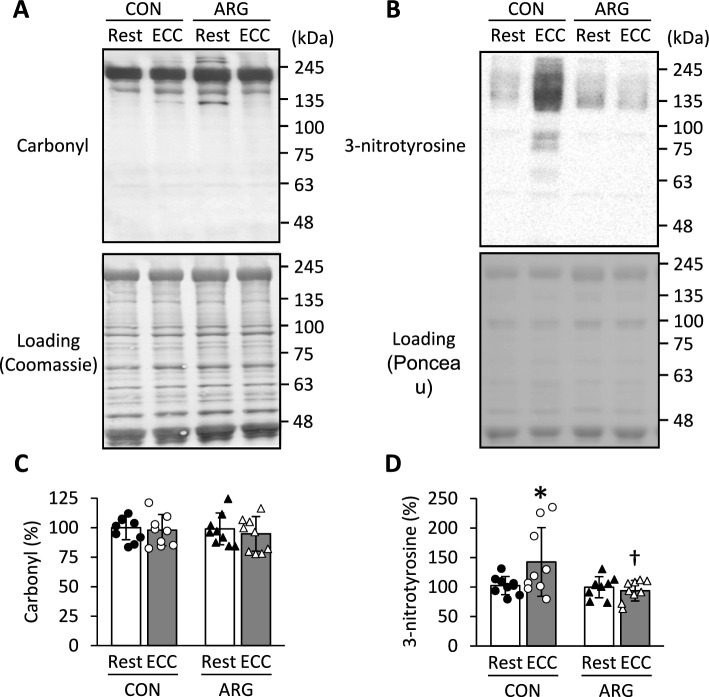
Effects of ARG ingestion and ECC induction on protein oxidation and nitration in tibialis anterior muscle. For the experimental protocols, see the legend of Fig. 1. **A** and **B** representative immunoblots of protein carbonyl and 3-nitrotyrosine, respectively. **C** and **D** carbonyl and 3-nitrotyrosine contents, respectively. Values are means ± SD (n = 9 for each group). For total protein quantification, the membrane was stained with Coomassie blue R or Ponceau S. Densitometric analysis of immunoreactive and total proteins was performed on all bands located on the membrane. The contents are normalized to total proteins. The results are expressed as percentages of the values in resting muscles from CON rats. *P < 0.05, vs. resting muscles within rats (rank transformation, followed by two-way repeated-measures ANOVA and Holm–Sidak post hoc tests); ^♰^*P* < 0.05, vs. ECC muscles from CON rats (rank transformation, followed by two-way repeated-measures ANOVA and Holm–Sidak post hoc tests). ARG, L-arginine; CON, control; ECC, eccentric contraction

Neither ARG ingestion nor ECC induction affected carbonyl content (Fig. 5A, C). On the other hand, a significant interaction was observed for 3-nitrotyrosine content, serving, as a marker of peroxynitrite formation (*P* = 0.043; Fig. 5B, D). In CON rats, 3-nitrotyrosine content in ECC muscles increased to 139% of that in resting muscles (*P* = 0.014); however, in ARG rats, 3-nitrotyrosine content did not differ between ECC and resting muscles. Additionally, 3-nitrotyrosine content in ECC muscles was significantly lower in ARG than in CON rats (*P* = 0.003).

## Discussion

Aligning with the results of this study, previous research on both rodents and humans demonstrated leukocyte invasion into ECC skeletal muscles (Paulsen et al. 2010; MacNeil et al. 2011). The main finding of the present study is the inhibitory effect of ARG ingestion on phagocyte invasion into ECC muscles and ECC-induced force depression, with the ECC-induced increases in CD68 content reduced approximately 4.4-fold. Given that ARG is a NO precursor, it is plausible that the observed effects of its ingestion are attributed to an increase in NO production.

Phagocytes play a critical role in clearing debris generated by ECC-induced mechanical and/or Ca^2+^ stress and in subsequent regeneration (Peake et al. 2017; Kanzaki et al. 2022). However, excessive invasion triggers severe inflammation due to superoxide, NO, and cytokines released by these pro-inflammatory cells (Peake et al. 2017). Therefore, ARG ingestion likely alleviates inflammation in ECC muscles, as evidenced by our observation of decreased 3-nitrotyrosine levels.

The mechanism by which ARG inhibits phagocyte invasion remains speculative. Gabriel et al. (2004) found that ARG infusion into a femoral vein before reperfusion reduced neutrophil adhesion to the vascular endothelium during ischemia–reperfusion. This reduction was reversed by coadministration of *N*(G)-nitro-L-arginine-methyl ester, a NOS inhibitor, indicating a NO-dependent effect. Notably, administration of 17β-estradiol, a female sex hormone, in males has been shown to prevent neutrophil invasion, but not macrophage invasion into ECC muscles (MacNeil et al. 2011). Moreover, neutrophil invasion precedes M1 invasion in ECC muscles (Peake et al. 2017). These findings suggest the distinct mechanisms underlying neutrophil and macrophage invasion. Hnia et al. (2008) proposed that ARG-derived NO may directly inactivate M1 located outside muscle cells. The inactivation of M1 macrophages could lead to a reduction in the number of M1 that infiltrates the muscle cells.

NOx concentration serves as an indicator of endogenously produced NO (Sakurai et al. 2013; Kanzaki et al. 2019). Our NOx results are consistent with previous findings showing ECC-induced increases in NO production (Sakurai et al. 2013). The elevated nitration observed in normal (CON) muscle likely arises as follows: (1) cell-infiltrating M1 release NO; (2) NO interacts with neutrophil-released superoxide; and (3) the interaction forms peroxynitrite, which nitrates proteins. Given the functional consequences of nitration on the myofibril (Dutka et al. 2011), it is likely to contribute to reduced force production in ECC muscles. In contrast, increased NO production in ARG-spared muscle may be attributed to increased NOS precursors (e.g., ARG). Based on previous findings (Gabriel et al. 2004; Hnia et al. 2008), ARG ingestion may attenuate neutrophil invasion and subsequent protein nitration.

Whether ECC shifts the muscle redox state to the oxidized side remains debatable. In one study, eccentric exercise brought about greater force depression but a minimal increase in reactive oxygen species production (Kamandulis et al. 2017). Conversely, another study reported that prior ingestion of dietary antioxidants inhibited force depression with ECC (Shafat et al. 2004). We observed no effect of ECC induction and ARG ingestion on protein carbonyl content, indicating that reactive oxygen species generation is not substantial. However, considering neutrophil and macrophage invasion into ECC muscle, no change in protein carbonyl levels might be expected, as most neutrophil-released superoxide forms peroxynitrite in normal ECC muscle (as mentioned earlier) and ARG blocks neutrophil invasion in ARG-spared ECC muscle.

In ECC muscles, calpains (Ca^2+^-regulated cysteine proteases) are activated by Ca^2+^ influx into muscle cells, cleaving various proteins involved in excitation–contraction coupling (Corona et al. 2010; Zhang et al. 2012; Kanzaki et al. 2017). Our previous study revealed that ARG ingestion attenuates ECC-associated proteolysis by decreasing calpain activation via *S*-nitrosylation of this protease (Kanzaki et al. 2018). ARG-induced calpain inactivation may also be mediated by inhibition of peroxynitrite production, as peroxynitrite indirectly activates calpains (Whiteman et al. 2004).

NOS exists in vivo as monomers and dimers. Only dimers can synthesize NO, whereas monomers generate superoxide using electrons from NADPH (Andrew and Mayer 1999). In an environment enhancing peroxynitrite production, the proportion of monomers and resulting superoxide production increase (Zou et al. 2002; Pandya et al. 2019). Therefore, an increased monomer proportion could be expected in ECC muscle. However, our eNOS results are contrary to this exception. Therefore, further research is needed to explore this discrepancy.

In summary, we show that ARG ingestion mitigates force depression and phagocyte invasion in ECC muscles. The unique feature of ECC muscles is to develop greater force with lower energy cost compared with muscles subjected to isometric or concentric contractions, allowing eccentric exercises to facilitate larger gains in strength and muscle mass. For competitive athletes, this feature is a great advantage. For the elderly, it will slow or prevent the progression of sarcopenia and reduce the risk of falls. However, ECC-related inflammation and prolonged force depression can impede training efficiency and individual motivation for exercise. The present findings suggest that ingestion of ARG or ARG-rich foods, such as soy protein isolate, would counteract these undesirable effects of eccentric exercise.

## Data Availability

We hope that our data will contribute to the advancement of human health and the improvement of performance in competitive sports.

## References

[CR1] Allen DG, Lamb GD, Westerblad H (2008) Skeletal muscle fatigue: cellular mechanisms. Physiol Rev 88:287–332 18195089 10.1152/physrev.00015.2007

[CR2] Allen DG, Whitehead NP, Froehner SC (2016) Absence of dystrophin disrupts skeletal muscle signaling: roles of Ca^2+^, reactive oxygen species, and nitric oxide in the development of muscular dystrophy. Physiol Rev 96:253–305 26676145 10.1152/physrev.00007.2015 PMC4698395

[CR3] Amanzada A, Malik IA, Blaschke M, Khan S, Rahman H, Ramadori G, Moriconi F (2013) Identification of CD68(+) neutrophil granulocytes in in vitro model of acute inflammation and inflammatory bowel disease. Int J Clin Exp Pathol 6:561–570 23573303 PMC3606846

[CR4] Andrew PJ, Mayer B (1999) Enzymatic function of nitric oxide synthases. Cardiovasc Res 43:521–531 10690324 10.1016/s0008-6363(99)00115-7

[CR5] Bradford MM (1976) A rapid and sensitive method for the quantitation of microgram quantities of protein utilizing the principle of protein-dye binding. Anal Biochem 72:248–254 942051 10.1016/0003-2697(76)90527-3

[CR6] Corona BT, Balog EM, Doyle JA, Rupp JC, Luke RC, Ingalls CP (2010) Junctophilin damage contributes to early strength deficits and EC coupling failure after eccentric contractions. Am J Physiol 298:C365–C376 10.1152/ajpcell.00365.2009 19940065

[CR7] Damoiseaux JG, Döpp EA, Calame W, Chao D, MacPherson GG, Dijkstra CD (1994) Rat macrophage lysosomal membrane antigen recognized by monoclonal antibody ED1. Immunology 83:140–147 7821959 PMC1415006

[CR8] Dutka TL, Mollica JP, Lamb GD (2011) Differential effects of peroxynitrite on contractile protein properties in fast- and slow-twitch skeletal muscle fibers of rat. J Appl Physiol 110:705–716 21030671 10.1152/japplphysiol.00739.2010

[CR9] Gabriel A, Porrino ML, Stephenson LL, Zamboni WA (2004) Effect of L-arginine on leukocyte adhesion in ischemia-reperfusion injury. Plast Reconstr Surg 113:1698–1702 15114131 10.1097/01.prs.0000117364.53547.0e

[CR10] Ghimire K, Altmann HM, Straub AC, Isenberg JS (2017) Nitric oxide: what’s new to NO? Am J Physiol 312:C254–C262 10.1152/ajpcell.00315.2016 PMC5401944 27974299

[CR11] Hnia K, Gayraud J, Hugon G, Ramonatxo M, Porte SDL, Matecki S, Mornet D (2008) L-arginine decreases inflammation and modulates the nuclear factor-κB/matrix metalloproteinase cascade in mdx muscle fibers. Am J Pathol 172:1509–1519 18458097 10.2353/ajpath.2008.071009 PMC2408412

[CR12] Hody S, Croisier JL, Bury T, Rogister B, Leprince P (2019) Eccentric muscle contractions: risks and benefits. Front Physiol 10:536 31130877 10.3389/fphys.2019.00536 PMC6510035

[CR13] Kamandulis S, de Souza LF, Hernandez A, Katz A, Brazaitis M, Bruton JD, Venckunas T, Masiulis N, Mickeviciene D, Eimantas N, Subocius A, Rassier DE, Skurvydas A, Ivarsson N, Westerblad H (2017) Prolonged force depression after mechanically demanding contractions is largely independent of Ca^2+^ and reactive oxygen species. FASEB J 31:4809–4820 28716970 10.1096/fj.201700019R

[CR14] Kanzaki K, Watanabe D, Aibara C, Kawakami Y, Yamada T, Takahashi Y, Wada M (2018) L-arginine ingestion inhibits eccentric contraction-induced proteolysis and force deficit via *S*-nitrosylation of calpain. Physiol Rep 6:e13582 29368397 10.14814/phy2.13582 PMC5789731

[CR15] Kanzaki K, Watanabe D, Aibara C, Kawakami Y, Yamada T, Takahashi Y, Wada M (2019) Ingestion of soy protein isolate attenuates eccentric contraction-induced force depression and muscle proteolysis via inhibition of calpain-1 activation in rat fast-twitch skeletal muscle. Nutrition 58:23–29 30273822 10.1016/j.nut.2018.06.025

[CR16] Kanzaki K, Watanabe D, Kuratani M, Yamada T, Matsunaga S, Wada M (2017) Role of calpain in eccentric contraction-induced proteolysis of Ca^2+^-regulatory proteins and force depression in rat fast-twitch skeletal muscle. J Appl Physiol (1985) 122:396–405 27979982 10.1152/japplphysiol.00270.2016

[CR17] Kanzaki K, Watanabe D, Shi J, Wada M (2022) Mechanisms of eccentric contraction-induced muscle damage and nutritional supplementations for mitigating it. J Muscle Res Cell Motil 43:147–156 35854160 10.1007/s10974-022-09625-1

[CR18] Klatt P, Schmidt K, Lehner D, Glatter O, Bachinger HP, Mayer B (1995) Structural-analysis of porcine brain nitric-oxide synthase reveals a role for tetrahydrobiopterin and L-arginine in the formation of an sds-resistant dimer. Embo J 14:3687–3695 7543842 10.1002/j.1460-2075.1995.tb00038.x PMC394443

[CR19] Lapointe BM, Frenette J, Cote CH (2002) Lengthening contraction-induced inflammation is linked to secondary damage but devoid of neutrophil invasion. J Appl Physiol (1985) 92:1995–2004 11960950 10.1152/japplphysiol.00803.2001

[CR20] Lomonosova YN, Shenkman BS, Kalamkarov GR, Kostrominova TY, Nemirovskaya TL (2014) L-arginine supplementation protects exercise performance and structural integrity of muscle fibers after a single bout of eccentric exercise in rats. PLoS ONE 9:e94448 24736629 10.1371/journal.pone.0094448 PMC3988069

[CR21] MacNeil LG, Baker SK, Stevic I, Tarnopolsky MA (2011) 17beta-estradiol attenuates exercise-induced neutrophil infiltration in men. Am J Physiol 300:R1443–R1451 10.1152/ajpregu.00689.2009 21368271

[CR22] Pandya CD, Lee B, Toque HA, Mendhe B, Bragg RT, Pandya B, Atawia RT, Isales C, Hamrick M, Caldwell RW, Fulzele S (2019) Age-dependent oxidative stress elevates arginase 1 and uncoupled nitric oxide synthesis in skeletal muscle of aged mice. Oxid Med Cell Longev 2019:1704650 31205583 10.1155/2019/1704650 PMC6530149

[CR23] Paulsen G, Crameri R, Benestad HB, Fjeld JG, Morkrid L, Hallen J, Raastad T (2010) Time course of leukocyte accumulation in human muscle after eccentric exercise. Med Sci Sports Exerc 42:75–85 20010127 10.1249/MSS.0b013e3181ac7adb

[CR24] Peake JM, Neubauer O, Della Gatta PA, Nosaka K (2017) Muscle damage and inflammation during recovery from exercise. J Appl Physiol (1985) 122:559–570 28035017 10.1152/japplphysiol.00971.2016

[CR25] Pizza FX, Peterson JM, Baas JH, Koh TJ (2005) Neutrophils contribute to muscle injury and impair its resolution after lengthening contractions in mice. J Physiol 562:899–913 15550464 10.1113/jphysiol.2004.073965 PMC1665528

[CR26] Sakurai T, Kashimura O, Kano Y, Ohno H, Ji LL, Izawa T, Best TM (2013) Role of nitric oxide in muscle regeneration following eccentric muscle contractions in rat skeletal muscle. J Physiol Sci 63:263–270 23606218 10.1007/s12576-013-0262-y PMC10717722

[CR27] Shafat A, Butler P, Jensen RL, Donnelly AE (2004) Effects of dietary supplementation with vitamins C and E on muscle function during and after eccentric contractions in humans. Eur J Appl Physiol 93:196–202 15309547 10.1007/s00421-004-1198-y

[CR28] Sloboda DD, Brown LA, Brooks SV (2018) Myeloid cell responses to contraction-induced injury differ in muscles of young and old mice. J Gerontol A Biol Sci Med Sci 73:1581–1590 29684112 10.1093/gerona/gly086 PMC6230214

[CR29] Voisin V, Sebrie C, Matecki S, Yu H, Gillet B, Ramonatxo M, Israel M, De la Porte S (2005) L-arginine improves dystrophic phenotype in mdx mice. Neurobiol Dis 20:123–130 16137573 10.1016/j.nbd.2005.02.010

[CR30] Whiteman M, Armstrong JS, Cheung NS, Siau JL, Rose P, Schantz JT, Jones DP, Halliwell B (2004) Peroxynitrite mediates calcium-dependent mitochondrial dysfunction and cell death via activation of calpains. FASEB J 18:1395–1397 15240564 10.1096/fj.03-1096fje

[CR31] Zhang BT, Whitehead NP, Gervasio OL, Reardon TF, Vale M, Fatkin D, Dietrich A, Yeung EW, Allen DG (2012) Pathways of Ca^2+^ entry and cytoskeletal damage following eccentric contractions in mouse skeletal muscle. J Appl Physiol 112:2077–2086 22461447 10.1152/japplphysiol.00770.2011 PMC3378392

[CR32] Zou MH, Shi C, Cohen RA (2002) Oxidation of the zinc-thiolate complex and uncoupling of endothelial nitric oxide synthase by peroxynitrite. J Clin Invest 109:817–826 11901190 10.1172/JCI14442 PMC150913

